# Assessment of early mitigation measures against COVID-19 in Puerto Rico: March 15-May 15, 2020

**DOI:** 10.1371/journal.pone.0240013

**Published:** 2020-10-14

**Authors:** Miguel Valencia, José E. Becerra, Juan C. Reyes, Kenneth G. Castro

**Affiliations:** 1 Data Management Operations for COVID-19 Legacy Systems, Puerto Rico Department of Health, San Juan, PR; 2 Graduate School of Public Health, University of Puerto Rico Medical Sciences Campus, San Juan, PR; 3 Department of Biostatistics and Epidemiology, Graduate School of Public Health, University of Puerto Rico Medical Sciences Campus, San Juan, PR; 4 Hubert Department of Global Health & Department of Epidemiology, Rollins School of Public Health, Atlanta, GA, United States of America; 5 Division of Infectious Diseases, Department of Medicine, School of Medicine, Emory University, Atlanta, GA, United States of America; University of Cyprus, CYPRUS

## Abstract

On March 15, 2020 Puerto Rico implemented non-pharmaceutical interventions (NPIs), including a mandatory curfew, as part of a state of emergency declaration to prevent the community transmission of the SARS-CoV-2 virus. The strict enforcement of this curfew was extended through May 25, with a gradual relaxation beginning on May 1. This report summarizes an assessment of these early mitigation measures on the progression of the COVID-19 pandemic in the island. From March 15 to May 15, 2020, 70,656 results of molecular (RT-PCR) tests were reported to the Puerto Rico Department of Health. Of these, 1,704 were positive, corresponding to 1,311 individuals with COVID-19 included in the study. We derived the epidemic growth rates (r) and the corresponding reproductive numbers (R) from the epidemic curve of these 1,311 individuals with laboratory-confirmed diagnosis of COVID-19 using their date of test collection as a proxy for symptoms onset. Through May 31, 2020, there were 143 COVID-19 associated deaths in Puerto Rico, for a case fatality risk of 10.9%. We compared the observed cases and deaths with Gompertz model projections had the mitigation measures not been implemented. The number of daily RT-PCR-confirmed cases peaked on March 30 (85 cases), showing a weekly cyclical trend, with lower counts on weekends and a decreasing secular trend since March 30. The initial exponential growth rate (r) was 15.87% (95% CI: 7.59%, 24.15%), corresponding to R of 1.82 (95% CI:1.37, 2.30). After March 30, the r value reverted to an exponential decay rate (negative) of -2.95% (95% CI: -4.99%, -0.92%), corresponding to R of 0.93 (95% CI: 0.86, 0.98). We estimate that, had the initial growth rate been maintained, a total of 6,155 additional COVID-19 cases would have occurred by May 15, with 211 additional COVID-19 deaths by May 31. These findings are consistent with very effective implementation of early NPIs as mitigation measures in Puerto Rico. These results also provide a baseline to assess the impact of the transition from mitigation to subsequent containment stages in Puerto Rico.

## Introduction

A novel coronavirus designated SARS-CoV-2 has been associated with coronavirus disease 2019 (COVID-19). The disease spectrum ranges from asymptomatic infections, mild-to-moderate influenza-like illness, pneumonia, severe acute respiratory distress, hyperinflammatory state, coagulopathy, to death [1–3]. First described in late 2019 in China, this novel virus rapidly spread for person-to-person and cases were identified in other provinces and 19 additional countries. By January 30, 2020, the World Health Organization (WHO) declared the COVID-19 outbreak a global health emergency [4]. With the rapid spread of this condition to a total of 113 countries, WHO Director General declared a global pandemic on 11 March 2020 [5].

In the absence of safe and effective vaccine or other therapeutic modalities against SARS-CoV-2, early measures in China included the implementation of non-pharmaceutical interventions (NPIs) aimed at temporizing the spread, illness, and deaths due to COVID-19 [6]. Similar interventions have been implemented in other countries; NPIs had previously been successfully used to mitigate the 1918–1919 influenza pandemic [7]. In the United States and Territories, the President and Governors declared health emergencies to implement NPIs, including orders to stay at home, travel restrictions, and closure of nonessential businesses [8].

Between March 9 and 13, 2020 the first confirmed COVID-19 cases were identified in Puerto Rico [9]. These were two tourists from Italy (ages 68 and 70), and a 71-year-old Puerto Rican with lymphoma and diabetes, whose relatives had recently traveled to Chicago, Illinois. Within the past 30 months Puerto Rico had experienced several major natural disasters, including hurricanes Irma and Maria in September 2017 with widespread interruption of the infrastructure for essential services. By late December 2019 and January 2020, the island experienced significant earth tremors and multiple aftershocks with extensive structural damage and housing instability in the Southwest, associated with limited health care access among vulnerable populations [10–12]. Against this backdrop, on March 15, 2020, the Governor of Puerto Rico issued an island-wide mandatory curfew as part of a state of emergency declaration closing public and private schools and universities, all nonessential businesses and public agencies, and implementing travel restrictions [13]. By then, five COVID-19 cases had been confirmed. The curfew, and its strict enforcement, were extended through May 25, with a gradual relaxation beginning on May 1. The curfew was accompanied by NPI mitigation measures which focused on identifying and isolating index COVID-19 cases and recommending a 14-day quarantine of contacts, in accordance with CDC guidelines. This report summarizes an assessment of these early mitigation measures on the progression of the COVID-19 pandemic in the island.

## Methods

All molecular diagnostic tests for SARS-CoV-2 infection (and COVID-19 case detection) were conducted using eight available FDA Emergency Use Authority (EUA)-approved real-time polymerase chain reactions (RT-PCR) test kits: 1) CDC 2019-Novel Coronavirus (2019-nCoV) Real-Time RT-PCR Diagnostic Panel (CDC); 2) Roche’s SARS-CoV-2Test; 3) LabCorp 2019 Novel Coronavirus (COVID-19) Test; 4) Quest SARS-CoV-2 rRT-PCR; 5) Panther Fusion SARS-COV-2 Assay; 6) Abbott RealTime SARS-CoV-2; 7) Xpert® Xpress SARS-CoV-2; and 8) ID NOW COVID-19. These diagnostic tests were conducted in approximately 900 laboratories throughout the island and processed by the Puerto Rico Department of Health (PRDH) Public Health Laboratory and five private reference laboratories, three (3) in Puerto Rico and two (2) in the United States. Test results were initially reported to the PRDH through an email address (labresult@salud.pr.gov), and later through a centralized electronic platform (BioPortal). From March 15 through May 15, 2020 results were reported to the PRDH, following CDC guidelines for COVID-19 case definitions both for cases and deaths [14]. All positive RT-PCR test results were de-duplicated to account for individuals who had more than one clinical specimen submitted for testing, and to accurately derive the number of individual COVID-19 cases over time (by date of sample collection). A de-identified public use time series database was used for analyses, and the project proposal was evaluated by the Human Research Subject Protection Office (HRSPO) at University of Puerto Rico Medical Sciences Campus. A determination was made, and documentation provided, by HRSPO that this type of secondary data analysis does not meet the federal regulations that define research with human subjects.

Using the epidemic curve, we compared the pre- and post- estimates of the epidemic growth rate (r) and its corresponding reproductive number (R) by March 30, two weeks after the mandatory curfew was implemented. Deaths were included for analysis through May 31 to account for the censoring effect in the registration of recent deaths. Given the 12-day phase shift between the two curves, a cohort approach for the estimation of the case fatality risk was adopted. As case and death records could not be individually linked (because COVID-19 deaths without confirmatory laboratory test were included, consistent with the CDC definition), the epidemic curve of confirmed COVID-19 cases was considered the population at risk to estimate the case fatality risk of COVID-19 associated deaths through May 31.

Results of a comprehensive retrospective cohort study of close contacts in Shenzhen, China, were used to impute the serial interval distribution (mean = 6.3 days, SD = 4.2) [15]. Computations were conducted in the R packages “incidence” and “epitrix” [16]. The reproduction numbers were calculated with the R package “incidence,” which fits two exponential models to incidence data, of the form: log(y) = r*t+b where ‘y’ is the incidence, ‘t’ is time (in days), ‘r’ is the growth rate, and ‘b’ is the origin. This package will fit one model by default, but can also fit two models on either side of a splitting date (typically the peak of the epidemic). Then, the R package “epitrix” converted the growth rate (r) into a reproduction number (R). The function r2R0 was used to transform a growth rate (r) into a reproduction number estimate (R), given a generation time distribution, with the corresponding credible intervals [17]. The averted number of confirmed COVID-19 cases, and the averted number of deaths associated with COVID-19, were estimated with a Gompertz model parametrized as y = N*exp(-exp(-a*(t-b))), using the nsl and forecast functions in R. Data through the peak of each curve were included to estimate projections [18].

## Results

The first seven COVID-19 cases in Puerto Rico had been routinely reported from March 9 through March 15, 2020. Among residents of Puerto Rico, a total of 70,656 results of molecular (RT-PCR) tests were reported to the Puerto Rico Department of Health between March 16 and May 15 (there were no cases reported on Sunday March 15). Of these, 1,704 (2.4%) were positive, corresponding to 1,311 individuals with COVID-19 included in the study. Demographically, 50.9% were male with a mean age of 46.0, and 49.1% female with a mean age of 45.5 (Table 1).

**Table 1:** Demographic characteristics of confirmed COVID-19 cases and deaths by age and gender.

	Confirmed Cases			Deaths		
Gender	n	Mean Age*	sd	n	Mean Age	sd
**F**	642	45.5	18.7	62	74.0	16.0
**M**	665	46.0	18.1	81	70.2	16.2
**All**	1307	45.8	18.4	143	71.9	16.2

*Four cases with missing age

**Fig 1:**
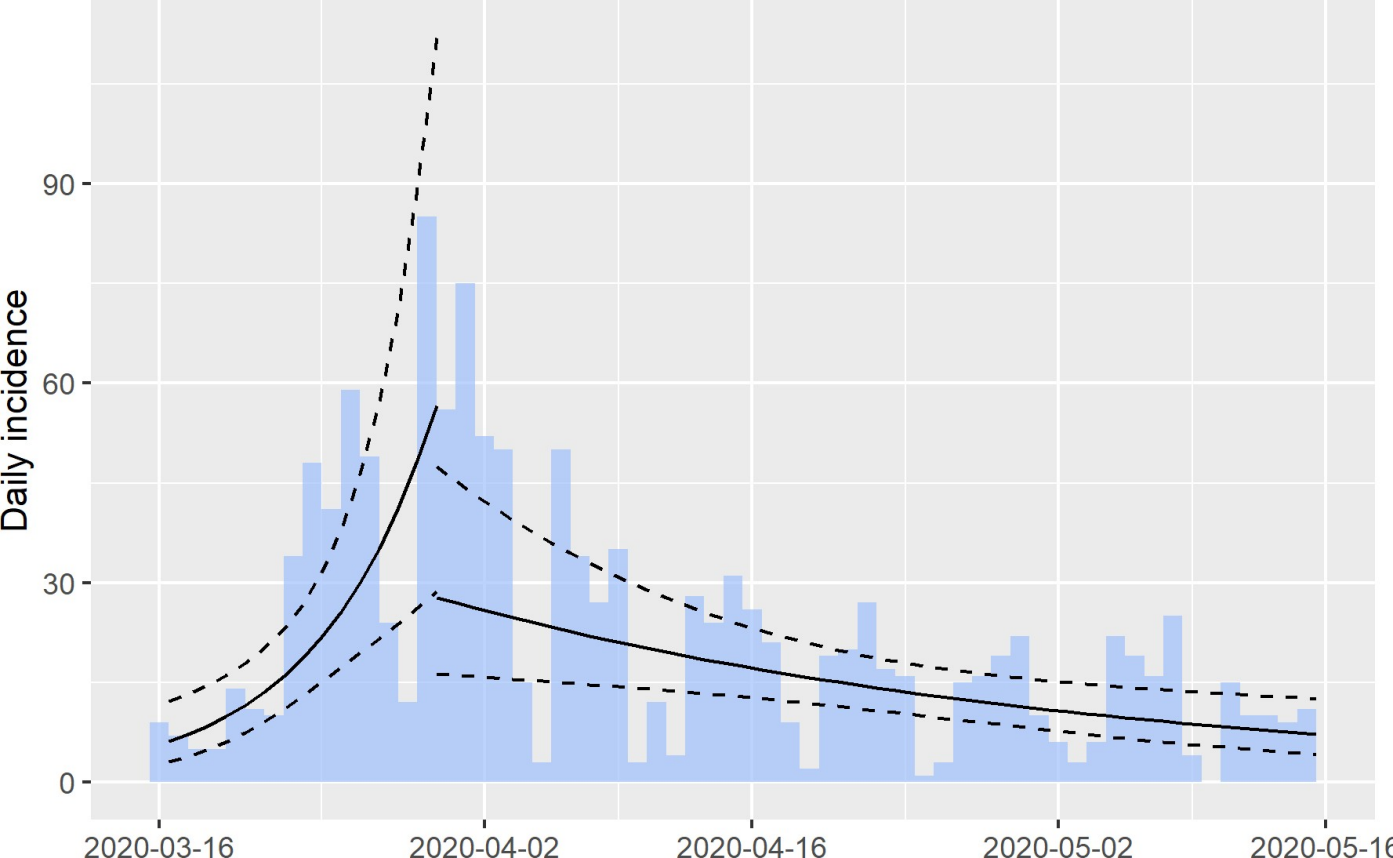
Epidemic curve of confirmed COVID-19 cases from March 15 –May 15. Epidemic curve of cases with log-linear regression line and 95% CI estimated before and after peak of 85 cases on March 30.

The number of daily RT-PCR confirmed cases peaked on March 30, showing a weekly cyclical trend, with lower counts on weekends and a decreasing secular trend since March 30 (Fig 1). Private laboratories close on weekends, leaving the PRDH Public Health Laboratory as the only lab serving the country; testing is reserved for symptomatic patients needing urgent care. The initial exponential growth rate (r) was 15.87% (95% CI: 7.59%, 24.15%), corresponding to R of 1.82 (95% CI:1.37, 2.30). After March 30, the r value reverted to an exponential decay rate (negative) of -2.95% (95% CI: -4.99%, -0.92%), corresponding to R of 0.93 (95% CI: 0.86, 0.98). Throughout the observation period, the total number of RT-PCR tests per day fluctuated but trended to a lower proportion of positive results (from 15.2% in the first two weeks to 0.3% in the last two weeks of the study period).

**Fig 2:**
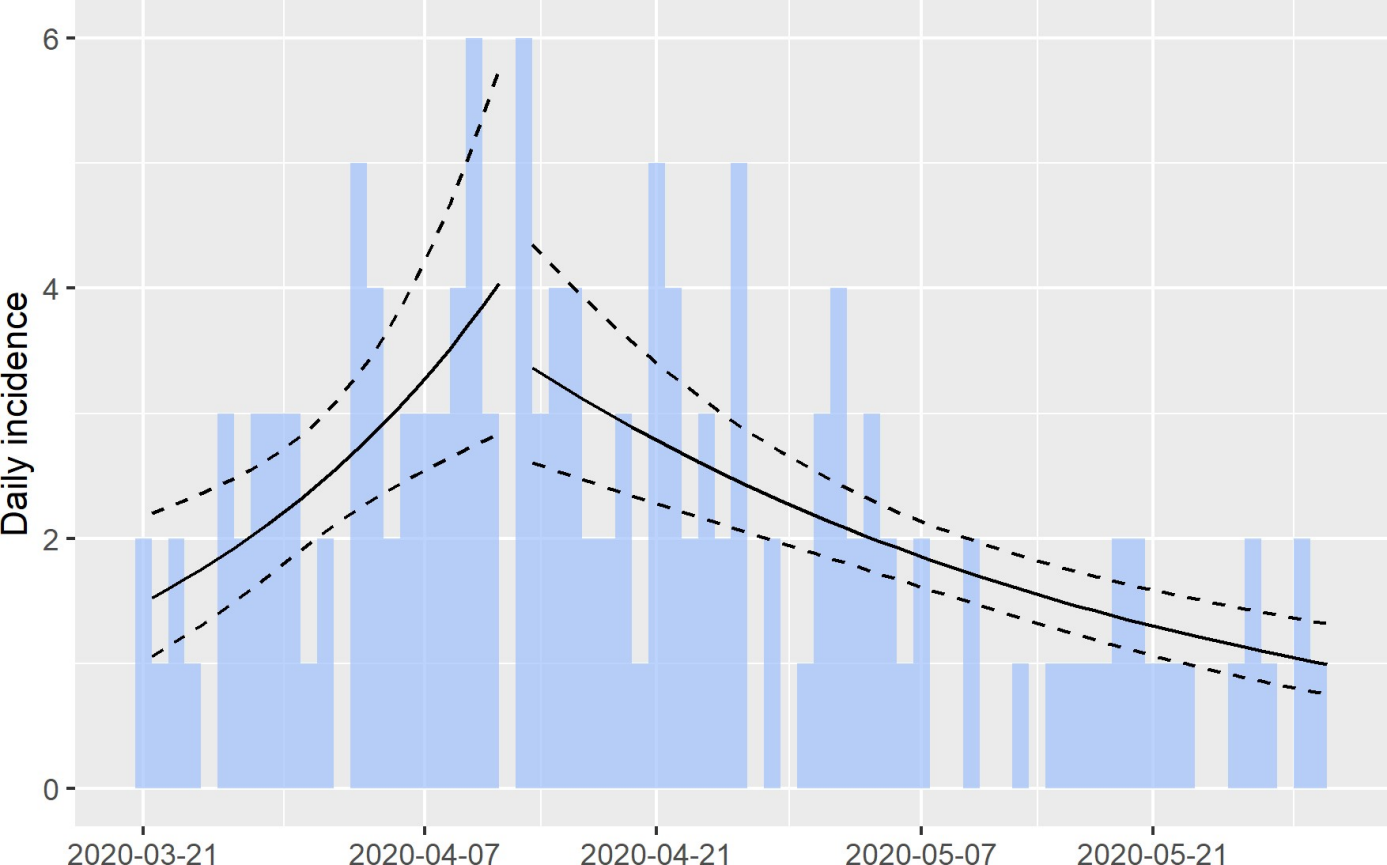
Distribution of COVID-19 related deaths. Epidemic curve of COVID-19 associated deaths with log-linear regression line and 95% CI estimated before and after peak of 6 deaths around April 12.

A total of 143 COVID-19 deaths were reported from March 15 through May 31, 2020. Of these, 126 (88.1%) had RT-PCR tests performed, 57 (45.2%) were positive (laboratory confirmed COVID-19 deaths) and 69 (54.8%) were negative (had COVID-19 as a probable cause of death); 81 (56.6%) were male with a median age of 70.2, and 62 (43.4%) were female with a median age of 74.0. (Table 1). Daily COVID-19 deaths peaked around April 12, 2020 (6 deaths on April 10 and on April 13). As illustrated in Fig 2, the initial exponential growth rate (r) of COVID-19 deaths was 4.64% (95% CI: 1.72%, 7.55%). After April 13, the r value reverted to an exponential decay rate (negative) of -2.54% (95%CI: -3.49%, -1.58%).

**Fig 3:**
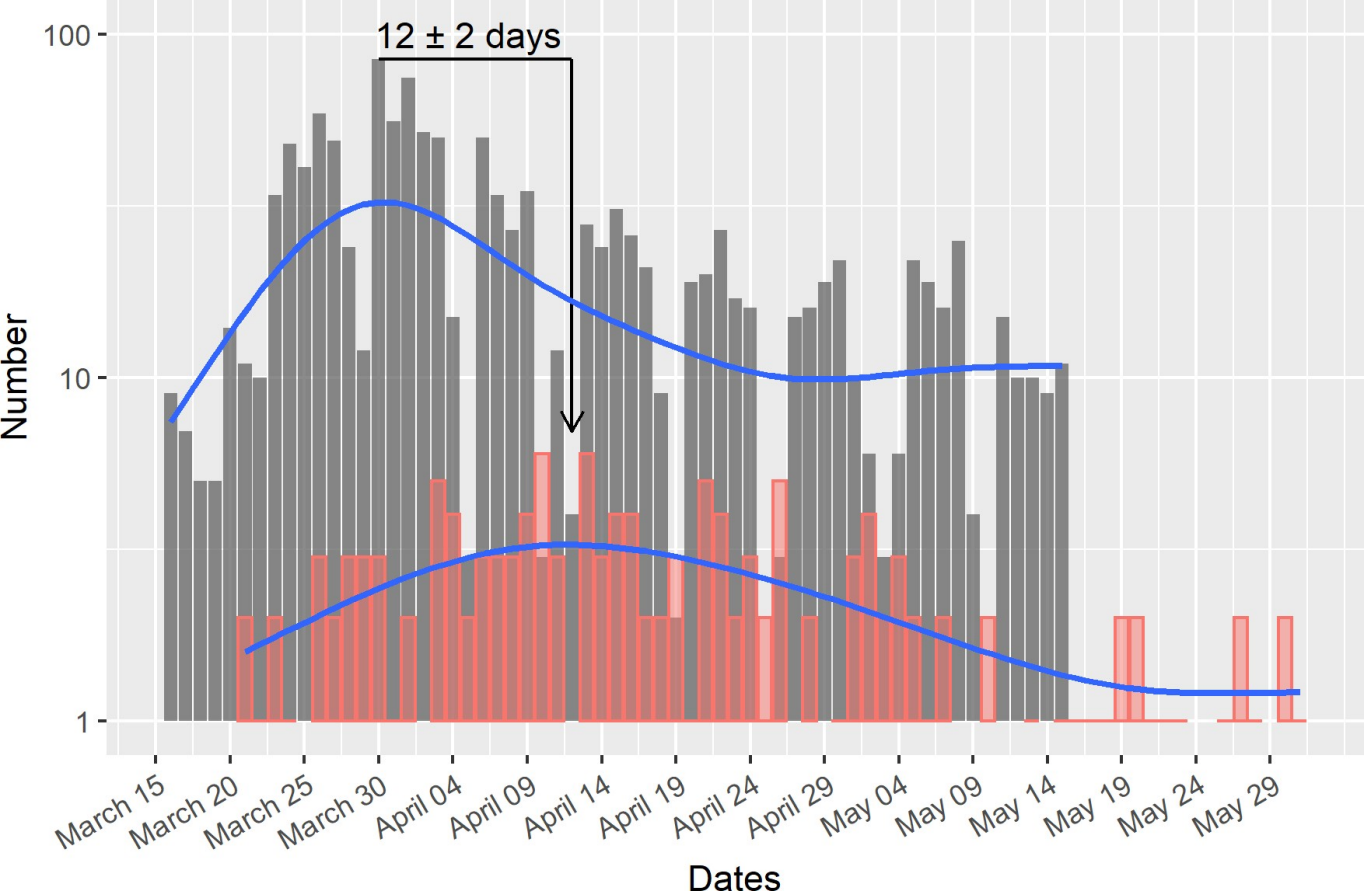
Epidemic curve of COVID-19 confirmed cases and deaths associated with COVID-19. Epidemic curve of COVID-19 cases (gray) and deaths associated with COVID-19 (red) showing a 12-day phase shift between their peaks (log scale). The blue curve shows their smoothed shapes.

The epidemic curve e of total confirmed COVID-19 cases through May 15, with its peak of 85 cases on March 30 (Fig 3), when laid over the distribution of deaths through May 31, with its peak around April 12 (6 deaths), shows a clear 12-day shift consistent with the expected delay between infection and death. Therefore, the case fatality risk can be estimated as 10.9% (143/1311), given that the population at risk for a COVID-19 related death would correspond to the COVID-19 cases included in the epidemic curve.

**Fig 4:**
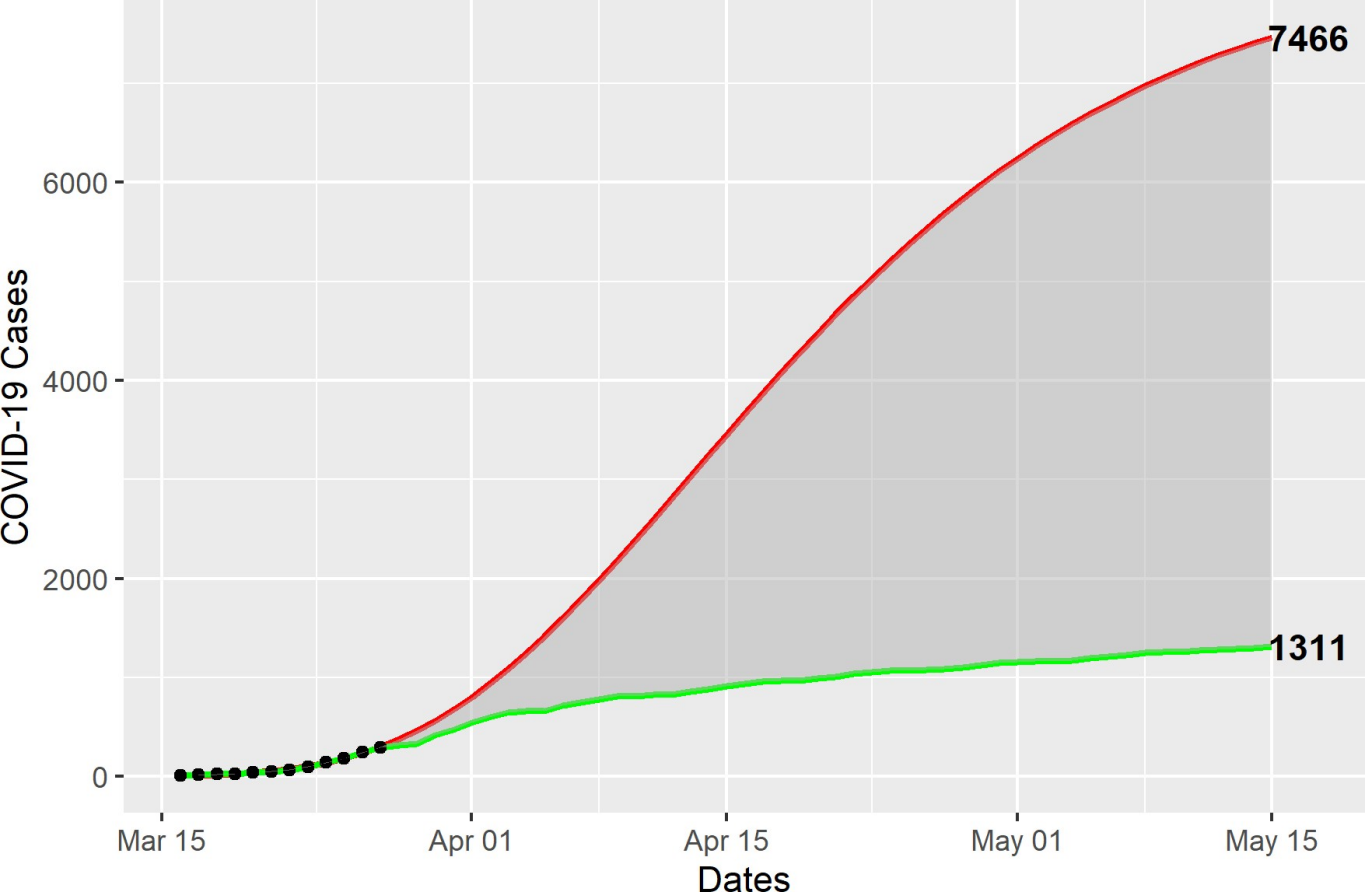
Gompertz model estimates of averted COVID-19 cases. COVID-19 confirmed cases (dots) from March 16 through March 28 (n = 292) were used to fit a Gompertz model (red line) to estimate the expected number of cases (n = 7,466), compared with the observed cases (green line), through May 15 (n = 1,311).

**Fig 5:**
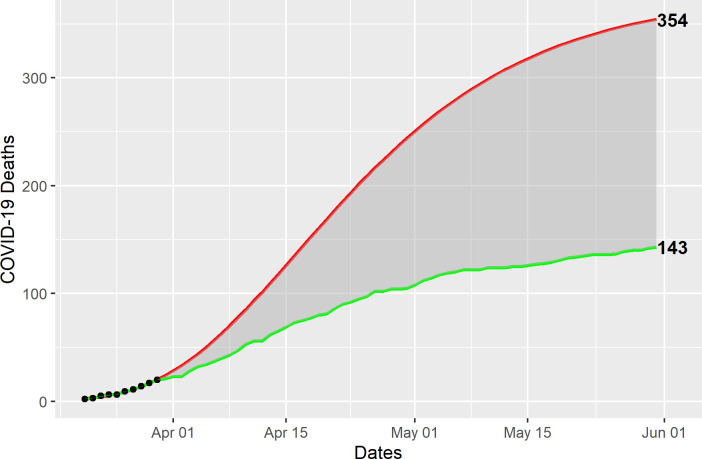
Gompertz model estimates of averted COVID-19 cases deaths. COVID-19 deaths (dots) from March 17 through March 26 (n = 20) were used to fit a Gompertz model (red line) to estimate the expected number of deaths (n = 354), compared with the observed deaths (green line), through May 31 (n = 143).

Using the Gompertz model, the estimated growth in COVID-19 cases would have reached a total of 7,466 by May 15, compared with the 1,311 observed (Fig 4). Similarly, the Gompertz model estimates 354 COVID-19 deaths by June 1, compared with 143 observed (Fig 5), for a total 6,155 COVID-19 cases and 211 deaths averted during the observation period. Given the relatively small number of observations, the predictive intervals are too wide to be meaningful.

## Discussion

Our findings are consistent with the use of NPIs as very effective early COVID-19 mitigation measures in Puerto Rico. It is likely that detection of COVID-19 cases remains under ascertained on the island, as suggested by the relatively high crude estimate of the case fatality risk of approximately 10%. However, this under ascertainment should not alter the estimates obtained from the epidemic curve provided the COVID-19 case ascertainment remained relatively constant throughout the study period. The observed decreasing trend in the proportion of RT-PCR positive tests lend support to this assumption. Furthermore, the mortality curve is consistent with a 2-week delay relative to the epidemic curve of the COVID-19 cases, as well as with hospital data reported elsewhere [2], confirming our findings about the effectiveness of the mitigation efforts. COVID-19 mortality may also be under ascertained in Puerto Rico.

We relied on relatively conservative Gompertz modelling to derive counterfactual estimates of the expected number of COVID-19 cases and deaths. Public health actions contributed to an estimated 6,155 averted COVID-19 cases and 211 averted deaths by May 31. These results show benefits associated with the early implementation of NPIs for COVID-19, with stay-at home orders, accompanied by mandated curfew and closure of non-essential services in a manner consistent with historical reports of benefits observed with similar use of NPIS in various U.S. cities during the 1918–1919 influenza pandemic [7]. For sustained benefit, a robust ongoing surveillance system will help identify incidence changes to inform policy decisions. In addition, these data serve as a baseline to assess the impact of the transition from mitigation to containment stages in Puerto Rico. We recommend that RT-PCR testing capacity to readily identify COVID-19 cases remain available for all symptomatic individuals in Puerto Rico, as well as for all essential workers, and that the public health infrastructure for case investigation and contact identification and tracing, accompanied by targeted isolation and quarantine, continue to be strengthened and scaled up to safeguard the gains observed to date.

## Supporting information

S1 File (R)

## Abbreviations

PRDH: Puerto Rico Department of Health
NPIs: non-pharmaceutical interventions
